# Privacy-Conducive Data Ecosystem Architecture: By-Design Vulnerability Assessment Using Privacy Risk Expansion Factor and Privacy Exposure Index

**DOI:** 10.3390/s25113554

**Published:** 2025-06-05

**Authors:** Ionela Chereja, Rudolf Erdei, Daniela Delinschi, Emil Pasca, Anca Avram, Oliviu Matei

**Affiliations:** 1 Department of Electrical, Electronics and Computer Engineering, Technical University of Cluj-Napoca, 400114 Cluj-Napoca, Romania; 2 R&D Department, Holisun, Str. Cărbunari nr. 8, 430397 Baia Mare, Romania

**Keywords:** privacy-first database systems, privacy by design, multi-layer data warehousing architecture, ecosystem architecture, cybersecurity, data protection, data vulnerability, privacy risk expansion factor, privacy exposure index, NewSQL

## Abstract

The increasing complexity of data ecosystems demands advanced methodologies for systematic privacy risk assessment. This work introduces two complementary metrics—the privacy risk expansion factor (PREF) and the privacy exposure index (PEI)—to evaluate how architectural decisions influence the exposure and distribution of sensitive data. Several representative use cases validate the methodology, demonstrating how the metrics provide structured insights into the privacy impact of distinct design choices. By enabling comparative analysis across architectures, this approach supports the development of privacy-first data ecosystems and lays the groundwork for future research on dynamic, AI-driven risk monitoring.

## 1. Introduction

In the era of data-driven ecosystems, the protection of personal and sensitive information has become a cornerstone of responsible digital infrastructure design. As global regulatory frameworks such as the General Data Protection Regulation (GDPR) [1] and the California Consumer Privacy Act (CCPA) [2] underscore the critical importance of protecting individual privacy, organizations are under increasing pressure to embed privacy-preserving principles into every layer of their data architectures [3]. However, despite these advancements, many privacy assessments remain narrowly focused on data content [4], often overlooking or under-representing the architectural and operational dimensions that significantly contribute to data exposure.

Modern digital ecosystems often span complex multi-tiered architectures [5,6] that include source databases, staging environments, analytical layers, and long-term archives. Within such environments, data replication is not only common but often necessary for redundancy, disaster recovery, and high availability. However, each additional copy, whether in the form of a backup, a distributed node, or a persistent cache, represents a potential point of vulnerability. These data instances may reside in different geographic locations [7], operate under varying jurisdictional controls [8], and be subject to different access policies and logging practices. Such heterogeneity introduces inconsistencies in the protection mechanisms applied across the data life cycle [9,10]. Moreover, the inherent complexity of such architectures presents significant challenges to balancing performance with security considerations [11,12,13].

The dynamic nature of data access, governed by diverse authentication and access control configurations, further complicates the ability to accurately assess privacy risks. While encryption and anonymization are vital tools, they are insufficient without a comprehensive view of how and where data proliferates within an ecosystem. The lack of standardized methodologies to evaluate the privacy implications of architectural decisions—such as data duplication, localization, and access scope—poses a significant gap in current risk models.

This paper addresses these overlooked dimensions by proposing a framework that quantifies architectural contributions to risk expansion. By treating replication, localization, containment, and access control as key factors, the model highlights the often overlooked increase in exposure resulting from the proliferation of multiple data instances across a system, instances that can significantly amplify privacy risks if not properly managed. Furthermore, evaluating the geolocation of such instances [14] introduces additional concerns related to data sovereignty [15,16] and jurisdictional constraints, which, although challenging to quantify, can have substantial implications in a comprehensive risk assessment. The goal is to support the development of ecosystems that are not only functionally robust but privacy conscious by design.

The remainder of this paper is organized as follows: Section 2.1 and Section 2.2 review the background and related literature; Section 3.1 outlines the objectives and scope of the proposed methodology; Section 3 details the methodological framework; Section 4 presents validation scenarios and representative use cases; Section 5 and Section 6 summarize the results, known limitations, and future work; and finally, Section 7 concludes the paper with a summary of key findings and future directions.

## 2. Materials and Related Work

### 2.1. Background and Definitions

The concept of privacy by design (PbD) has emerged as a cornerstone in the development of modern data systems, advocating the integration of privacy and data protection principles from the earliest stages of system architecture and design [17,18]. As data ecosystems grow in complexity, including distributed infrastructures, multi-tiered storage, and cloud-based services, the potential for data exposure and misuse increases significantly [19,20,21,22]. PbD [23,24] emphasizes proactive rather than reactive measures, embedding safeguards directly into the technological framework rather than relying solely on post-deployment controls. This approach not only ensures compliance with regulatory requirements such as GDPR [1] and the Health Insurance Portability and Accountability Act (HIPAA) [25], but it also fosters trust, resilience, and accountability within digital infrastructures. Consequently, designing with privacy as a foundational principle is no longer optional but essential to ensure ethical, sustainable, and secure data-driven innovation. The PbD principles also seek to accommodate all legitimate interests and objectives without jeopardizing data privacy. However, each design choice made in the architecture of such an ecosystem must achieve the right balance between data functionality and availability, along with the measure of protection [26]. Changes in either of these settings will have an impact on the other and the overall infrastructure of a system [27].

A key implementation of privacy by design is the principle of data minimization [28], which entails collecting and retaining only the data strictly necessary for a specific purpose. This practice significantly reduces the vulnerability of the system by limiting the volume and sensitivity of stored data, thereby shrinking the attack surface. Embedded in legal frameworks like GDPR [1] and widely recognized in privacy engineering methodologies, data minimization not only supports compliance but reinforces the proactive ethos of privacy by design by limiting risk exposure from the outset [17,29].

However, in practice, achieving data minimization is often constrained by the architectural requirements of modern data systems. Data warehousing for analytics, machine learning pipelines, large language models (LLMs), and distributed database architectures frequently requires data duplication, prolonged retention, and complex storage hierarchies [30]. These designs, while optimizing performance and insight generation, can inadvertently conflict with minimization goals, making it critical to balance utility with privacy-preserving constraints [31,32].

Privacy-enhancing technologies (PETs) have become essential for safeguarding data in modern ecosystems. Techniques such as differential privacy, which injects statistical noise to obscure individual-level information [33], and homomorphic encryption, which allows computation on encrypted data without decryption [34], offer robust protection while maintaining data utility. Secure multiparty computation (SMPC) allows multiple entities to jointly compute outcomes without revealing their input [35], while trusted execution environments (TEE) protect sensitive computations in hardware-isolated environments [36]. Federated learning presents an alternative to centralized data processing by training machine learning models locally on edge devices and only sharing model updates [37], significantly reducing raw data exposure. Additional techniques such as synthetic data generation and pseudonymization/anonymization also help mitigate re-identification risks. However, despite their strengths, these PETs focus on protecting the content of the data and do not address the inherent vulnerability posed by its existence and replication. Unauthorized access to any copy of the data—whether a backup, distributed node, or replica—could potentially bypass content-level protections. Therefore, every additional copy increases the exposure surface of the system, highlighting the critical importance of evaluating architectural decisions that influence data duplication and localization.

### 2.2. Related Work

Several prior efforts have aimed to assess data protection within system architectures. Sion et al. [38] introduce a GDPR-aligned framework that provides an architectural perspective for data protection by design, emphasizing system responsibilities and data flows to help developers identify where privacy controls are necessary within the infrastructure. However, their approach is largely qualitative and lacks quantitative metrics for assessing architectural vulnerability or propagation of risk due to data replication or geopolitical concerns. Similarly, Morali et al. [39] propose a model-driven method for the assessment of confidentiality risk in architectures, focusing on identifying the propagation of threats across components and assets. Although more formalized, this model does not account for the number of data copies, geographic data dispersion, or the varying access scopes that can significantly impact exposure. Antignac and Le Métayer [40] present a formal approach to reasoning about privacy architectures, particularly focusing on principles such as data minimization and integrity, but they focus on abstract system guarantees rather than concrete data infrastructure or physical exposure risks. Wagner and Boiten [41] advance the discussion of privacy risk metrics, aiming to establish general-purpose privacy indicators, though these remain mostly content and policy centric, overlooking structural and operational aspects of system architecture.

Senarath et al. [42] emphasize the importance of minimizing unnecessary data use in accordance with the GDPR’s data minimization principle; however, they do not consider the impact of data replication or geographic distribution on privacy risks. Similarly, De and Le Métayer [43] focus on informing users about data risks and access governance, while Bakar et al. [44] advocate for quantifying risks related to developer access permissions, but neither addresses how architectural decisions, such as data replication or localization, affect overall risk. Cao et al. [45] analyze privacy risks in continuous data streams considering temporal correlations, but they overlook the influence of data sharing and replication across distributed systems. Giomi et al. [46] propose a framework for quantifying residual privacy risks in synthetic data, but their focus is limited to synthetic environments and does not extend to real-world infrastructures where data replication and access control are critical. Eldin et al. [47] present a risk scoring model for open data, but they do not account for how architectural factors influence privacy risks.

In the context of the Internet of Things (IoT) and event-driven systems [48,49], existing frameworks have made significant strides in identifying privacy challenges [50,51]. However, they often fail to address the architectural dynamics unique to sensor-based infrastructures. For example, Alwarafy et al. [52] provide a comprehensive survey of edge-assisted IoT environments and identify architectural vulnerabilities related to data localization and replication, but their treatment remains primarily categorical rather than quantitative. Similarly, Chakraborty et al. [53] introduce privacy-preserving mechanisms for quality assessment of IoT time series data, but they do not model how sensor data, often replicated across edge nodes, cloud layers, and storage silos, contribute to the expansion of privacy risk or how contextual architectural exposure (including geopolitical dispersion and access scope variation) further impacts the exposure of data.

While existing methodologies address aspects of privacy risk [54] and attack surface analysis, to the best of our knowledge, there is no established model that directly quantifies the expansion of the privacy attack surface resulting from architectural storage configurations for data storage systems in a formalized metric-based approach. Traditional attack surface metrics, such as those proposed by Manadhata and Wing [55], focus on quantifying system exposure through entry points, channels, and data types, primarily in software systems. In the realm of privacy risk assessment, frameworks like the NIST Privacy Risk Assessment Methodology (PRAM) [56] offer structured approaches to evaluate privacy risks. However, they do not specifically quantify the impact of architectural storage configurations on the expansion of risk.

To address these limitations, we propose the privacy risk expansion factor (PREF), a quantitative method that evaluates how architectural decisions affect the expansion and propagation of privacy risks across a system. In addition, by combining PREF with the vulnerability score (VS) from our previous work [57,58], which assesses data vulnerability, we present the privacy exposure index (PEI). This new index offers a comprehensive metric for evaluating both content-based and architecture-induced vulnerabilities in an effort to move closer to a generalized privacy exposure assessment framework, inspired by the principles of the Common Vulnerability Scoring System (CVSS) [59,60].

## 3. Methodology

### 3.1. Framework Objectives

This section defines the conceptual boundaries of the proposed privacy risk expansion factor (PREF) methodology. PREF is focused on quantifying the expansion of privacy risk that arises directly from architectural decisions within data systems. It captures how structural elements such as data replication, geographic location, access control configurations, and persistence of data in backups or logs contribute to the overall spread and durability of data exposure. For example, even after a user deletes their data at the application level, residual copies may continue to exist in system logs, persistent caches, or off-site backups, maintaining potential privacy risks. PREF treats these risks as inherent to the architecture itself, independent of the specific data content. In other words, even as the datasets change, the risk of structural exposure remains stable unless the system architecture is modified. By isolating and measuring this architecture-induced expansion of risk, PREF establishes a foundation for more comprehensive and infrastructure-aware privacy assessments, ultimately supporting efforts towards a standardized evaluation framework for data vulnerabilities.

The privacy risk expansion factor (PREF) is not intended to completely replace traditional security assessments at the data content or even architectural level. Instead, it complements such evaluations by quantifying how architectural decisions, captured through governance and access, retention and disposal, and secondary exposure metrics, contribute to the expansion of data exposure risk. The innovation introduced by PREF lies in its ability to model how variations in these dimensions amplify or attenuate the overall exposure, providing a structured way to assess how changes in architecture impact the exposure risk, regardless of the inherent sensitivity of the data.

To provide a more holistic and practical measure of privacy vulnerability, we propose the combined assessment of PREF and the previously introduced vulnerability score (VS). The VS captures the intrinsic sensitivity and exposure risk of the data itself and is explicitly designed to accommodate a variety of data types, including structured, semi-structured, and unstructured data. The proposed resulting composite metric, named the privacy exposure index (PEI), integrates both the data-centric and architecture-induced dimensions of risk.

By uniting PREF and VS into the PEI, this framework enables a more standardized, transparent, and adaptable methodology for assessing privacy vulnerabilities in complex data systems. Crucially, the framework is storage agnostic. It is applicable across relational databases, distributed file systems, NoSQL or NewSQL databases, and cloud native storage infrastructures. As the evaluation is grounded in system behavior and data properties rather than being tied to any specific storage technology, PEI provides a consistent basis to compare privacy exposure across diverse implementation environments.

### 3.2. Privacy Risk Expansion Factor (PREF)

We propose the introduction of the privacy risk expansion factor (PREF), which is designed to quantify the amplification of privacy risk within a data layer due to architectural design choices for the data storage system. The Privacy risk expansion factor (PREF) is defined by quantifying a series of architectural privacy metrics to be assessed for each data copy. In this context, a data copy refers to any additional instance of a dataset within a storage system, including but not limited to distributed or high-availability replicas, backups, persistent caches, disaster recovery copies, or any other form, complete or partial, that can be accessed independently and contains data potentially subject to exposure.

We proposed the following metrics, grouped into three main categories, based on the nature of the risk they capture:

1. Governance and Access Metrics capture how the storage architecture controls and restricts access to data, as well as the geopolitical implications of its location:
  (a) $A_{i}$ (Access Control Scope): This represents the extent and granularity of access control mechanisms applied to the *i*-th data copy. Potential values are [1–2], where a lower value means strictly limited access with fine-grained roles and strong policies with automated auditing, and a higher value indicates broad or poorly enforced access rights (see Appendix A).
  (b) $G_{i}$ (Geopolitical Location Risk): This reflects the privacy and regulatory risk associated with the physical or legal location of the *i*-th data copy. Potential values are [1–2], where low values represent the introduction of minimal risk relative to expected geopolitical and legal constraints, while higher values indicate increased potential for extraterritorial access, weak regulatory oversight, or misalignment with the required data localization standards (see Appendix B).
2. Retention and Disposal Metrics evaluate the policies and technical mechanisms for governing data lifespan and erasure:
  (a) $R_{i}$ (Retention and Lifecycle Policy Compliance): This captures whether a data copy is governed by a defined and automatically enforced retention policy. Potential values are [0–1], where higher values indicate weak, absent or manual policies leading to prolonged exposure (see Appendix C).
  (b) $D_{i}$ (Data Erasure Resistance): This represents how difficult it is to fully remove or delete the *i*-th data copy. Potential values are [0–1], where low values mean an easily erasable copy without the ability to recover data after deletion, and higher values indicate high persistence and/or potential to retrieve the data after deletion (see Appendix D).
3. Secondary Exposure Metrics consider indirect or auxiliary sources of privacy leakage outside the primary data store:
  (a) $L_{i}$ (Log Accessibility Risk): This indicates the exposure of logs of the *i*-th copy. Potential values are [0–1], where low values reflect no exposed logs, and high values represent logs that are accessible, poorly secured, or maintain traces of deleted data (see Appendix E).

Privacy risk expansion factor (PREF) for a single data layer is calculated using the relation shown in Equation (1)

(1) $$\mathrm{PREF}=\sum_{i=1}^{n}\left(G_{i}\cdot A_{i}\cdot \left(1+R_{i}+D_{i}+L_{i}\right)\right)$$

where

- *n* is the number of distinct data copies in the layer (e.g., database nodes, backups, distributed replicas).

The formulation of the privacy risk expansion factor (PREF) (Equation (1)) adopts a multiplicative structure to reflect the compounding nature of architectural privacy risks. Specifically, the product of $G_{i}$ and $A_{i}$ is used to model the interaction between geopolitical and access-related vulnerabilities. This formulation reflects that both geopolitical location and access control are primary determinants of systemic exposure risk [61,62,63,64] and thus carry greater weight in the final PREF score. A higher value in either metric not only indicates direct risk, but also amplifies the influence of secondary factors such as retention, erasure resistance, and log exposure whose impact is conditional on the accessibility of the data copy and its legal jurisdiction [14]. In scenarios where either governance or access is highly restrictive, the overall risk introduced by that copy should remain relatively low; hence, multiplication serves to attenuate risk when one component is well managed and to amplify it when both are weak.

Alternative aggregation strategies were considered during the design of PREF. Additive models, including simple or weighted summation, were found to inadequately represent situations where multiple weak controls should jointly elevate risk. In additive systems, a strong score in one dimension may offset a poor score in another, which contradicts the intended semantics of compounded vulnerability. For example, low geopolitical risk should not neutralize the consequences of overly permissive access.

Normalization-based approaches were also evaluated, but such models tended to reduce the interpretability and transparency of the aggregated metric, especially when assessing the marginal impact of individual architectural changes.

Thus, the multiplicative formulation was selected for its ability to preserve the independent contribution of the metrics while sensitively scaling the final PREF score in proportion to the systemic weakness across the proposed dimensions. This approach aligns with established practices in risk modeling, where multiplicative terms are used to capture interdependencies and conditional escalation of risk. As a result, we propose that the scales of the governance and access metrics range from 1 to 2 rather than 0 to 1 to ensure that their multiplicative role in the PREF formulation appropriately reflects their amplifying impact on overall risk (see Appendix A and Appendix B). This choice avoids nullifying the compounded risk in cases where one factor is well managed (i.e., near zero), but the other is critically weak. In contrast, the retention, disposal and secondary exposure metrics are assigned values between 0 and 1, as they serve to incrementally adjust total risk based on more localized or secondary vulnerabilities, which are conditionally affected by governance and access posture (see Appendix C, Appendix D and Appendix E).

For a clear overview of the entire ecosystem’s risk exposure, we also propose the calculation of an ecosystem privacy risk expansion factor ($PREF_{E}$), which is determined as the sum of all individual layers’ PREF scores within the system (Equation (2)). While the calculation of PREF for a single layer helps highlight the expansion of privacy risk, particularly when the resulting value exceeds 1, indicating risk amplification beyond baseline, we propose $PREF_{E}$ as a means to evaluate the cumulative risk across all persistent storage components in a data ecosystem. By structuring the calculation of $PREF_{E}$ through a matrix that includes each layer and its corresponding PREF value, this approach offers a valuable diagnostic tool to identify and troubleshoot components that contribute disproportionately to overall exposure.

(2) $$PREF_{E}=\sum_{L=1}^{m}PREF_{L}$$

Here,

- *m* is the total number of data layers in the ecosystem architecture.
- $PREF_{L}$ is the privacy risk expansion factor calculated for the *L*th layer in the architecture, as defined in the layer-level calculation (see Equation (1)).

Upon analysis, it becomes evident that the resulting PREF and EPREF values lack a defined upper bound. This comes from the architectural flexibility that allows virtually unlimited replication of data across different storage components or services. As such, PREF values cannot be meaningfully normalized or mapped onto a fixed scale (e.g., 0 to 100), since there is no theoretical limit to the number of data copies an architecture may accommodate.

In practice, any addition of data copies or replication layers for performance, redundancy, or backup purposes will proportionally increase the PREF score. Likewise, architectural decisions that weaken privacy safeguards (e.g., applying less-restrictive access control or exposing logs) will also amplify the PREF value. This model allows for a more precise assessment of architectural decisions by factoring in both the quantity and the individual characteristics of each data copy within a specific layer as well as the overview of the entire ecosystem.

### 3.3. Ecosystem Vulnerability Score

Proposed in previous work [58], the vulnerability score (VS) is a metric that quantifies the inherent privacy risk associated with a given data layer. It reflects the extent to which the data is susceptible to exposure or misuse, serving as an indicator of its overall vulnerability within a system.

As defined in our previous research [58], the assessment of data vulnerability is divided into two primary categories: data persistence metrics and data contents metrics. Data persistence metrics evaluate the risk associated with the method of data storage. The encoding level (ENCD) measures whether data is anonymized or not, with values ranging from 1 to 2, where 1 indicates anonymized data, and 2 denotes non-anonymized data. Encryption (ENCR) assesses the strength of the encryption used; values also range from 1 to 2, where 1 represents strong, secure encryption methods and 2 represents weak or absent encryption (see Appendix F). Masking status (MASK) evaluates whether the data field is masked, using the same 1 to 2 scale—1 for fields that are masked according to a correctly applied secure policy, and 2 for unmasked fields or improper policies.

Data contents metrics assess how sensitive and exploitable the stored data is. Exploitability (EXPL) refers to how easily a piece of data can be leveraged or misused, with values from 1 to 2, where more exploitable data receives a higher score (see Appendix G). The personal information data metric (HPI) identifies the presence of personal identifiable information (PII), with values of 0 or 1. Sensitive/secret information (HSI) reflects whether the data contains confidential content, with values from 0 to 1. Nested sensitive data (HNS) refers to sensitive information that is embedded within semi-structured data fields, and it is also rated from 0 to 1 based on its complexity. Finally, the unstructured sensitive data (HUS) metric captures the presence of sensitive content in unstructured formats, and it is similarly scored between 0 and 1. To maintain clarity and avoid redundancy, the explanations of these sub-metrics have been kept concise in this manuscript. For comprehensive definitions, examples, and empirical justification of each component, we refer the reader to our previous work [58], where the development and rationale behind the vulnerability score and the ecosystem vulnerability score are detailed.

The following equations describe how the vulnerability risk of a specific data layer (Equation (3)) and the total ecosystem score (Equation (4)) are calculated:

(3) $$VS_{L}=\sum_{i=1}^{n}\left(ENCD_{i}\cdot ENCR_{i}\cdot MASK_{i}+EXPL_{i}\cdot \left(1+HPI_{i}+HSI_{i}+HNS_{i}+HUS_{i}\right)\right)$$

(4) $$VS_{E}=\sum_{L=1}^{m}VS_{L}$$

Here,

- $VS_{L}$ represents the vulnerability score of the data storage layer;
- $VS_{E}$ represents the ecosystem vulnerability score accounting for all data layers included;
- *n* represents the number of fields in the dataset;
- *m* represents the number of data storage layers in the ecosystem.

A higher vulnerability score indicates increased vulnerability of the data stored within a specific data layer.

### 3.4. Privacy Exposure Index (PEI)

The privacy exposure index (PEI) represents the overall risk of exposure for sensitive data by capturing the compounded effect of two critical dimensions: the vulnerability of the data content and the exposure introduced by the system architecture that stores or processes it. PEI is formally defined for a given persistent data layer as

(5) $$PEI_{L}=PREF_{L}\cdot VS_{L}$$

where

- $PREF_{L}$ represents the privacy risk expansion factor for a single data layer;
- $VS_{L}$ represents the vulnerability score for the same data layer.

PEI is introduced as a single, unified metric that enables a structured assessment of compounded privacy risk by integrating both intrinsic data sensitivity and extrinsic architectural exposure. In developing this formulation, several aggregation approaches were considered to model the interaction between PREF and VS, including additive and weighted average models. However, due to the nature of the relationship between content-based vulnerability and system-induced exposure, which is not simply cumulative but mutually amplifying, a multiplicative formulation was selected.

This multiplicative model captures a core principle in risk modeling: When two independent but necessary components of risk are present, their interaction escalates the overall threat level disproportionately. For example, highly sensitive data (high VS) stored in a highly exposed or weakly governed architecture (high PREF) yields a significantly higher overall privacy risk than either factor alone would suggest. In contrast, additive models may fail to adequately reflect this escalation, especially in high-risk scenarios. While additive models are generally easier to interpret and normalize, they under-represent the compounding nature of exposure.

The total ecosystem PEI is calculated as

(6) $$PEI_{E}=\sum_{L=1}^{m}PREF_{L}\cdot VS_{L}$$

where

- $PREF_{L}$ represents the privacy risk expansion factor for the data layer;
- $VS_{L}$ represents the vulnerability score of the same data layer;
- *m* represents the number of data storage layers in the ecosystem.

It is important to be able to calculate the scores at each data layer as well as for the entire ecosystem, as each layer can have a separate configuration in place for the replication, distribution, and retention of the data stored. Assessing the scores for each layer and as a total will indicate both where the system has the most risk exposure and data vulnerability, as well as how it compares overall with a different type of architecture design that includes other settings.

The natures of the privacy risk expansion factor (PREF), vulnerability score (VS), and privacy exposure index (PEI) are inherently dynamic, reflecting the evolving characteristics of both data and architectural design choices. As data schemas change and system configurations adapt, so too must the metrics used to assess risk. Future research should explore how these assessments can be partially or fully automated, for instance, through the use of data contracts or other declarative system specifications to evaluate VS metrics. Additionally, system configurations could be leveraged to dynamically compute and adjust PREF metrics in real time. The integration of machine learning models for automatic risk evaluation and continuous monitoring also represents a promising direction to enhance scalability and responsiveness.

### 3.5. Interpretation of VS, PREF, and PEI

The vulnerability score, by design, responds proportionally to the number and sensitivity of fields in the data storage layer. As data accumulates, particularly data deemed personally identifiable or sensitive, the vulnerability score naturally increases. This property highlights the importance of selective data minimization and careful schema design as proactive privacy strategies. Rather than treating all data equally, system architects are encouraged to evaluate the necessity and granularity of stored fields in order to manage vulnerability early in the pipeline.

While the VS metric reflects what is stored, the PREF metric captures how that data is exposed architecturally. A critical insight is that high PREF values can emerge even when the underlying data is not inherently high risk (i.e., has a low VS). This allows system designers to identify problematic architectural patterns that may unnecessarily increase surface exposure and operational complexity (such as excessive duplication or poorly governed storage copies). Therefore, PREF can act as a pressure point to reevaluate deployment decisions, such as whether all data copies need to persist across regions or services.

Combined, VS and PREF offer complementary perspectives that reflect two critical dimensions of privacy risk. PEI unifies both content-level and architecture-level risks into a single, interpretable value that represents the overall likelihood and impact of exposing sensitive or high-value data. This holistic view is essential for designing systems that are both privacy-aware and operationally sound.

Importantly, because neither metric is bounded to a fixed upper limit, they scale in ways that reflect real-world risk accumulation. Adding additional sensitive fields increases VS; creating additional exposed instances increases PREF. This characteristic reinforces their utility in dynamic environments, where changes to either the schema or architectural model can be tracked longitudinally to identify growing privacy risk before it becomes unmanageable.

## 4. Use Cases and Validation

To validate the proposed methodology, we employ it in two reduced-complexity empirical cases by proposing and evaluating several distinct data architecture designs. This approach enables a comparative analysis that illustrates how PREF, VS, and PEI metrics contribute to the assessment of privacy risks across different architectural choices while maintaining the datasets across deployment models. As PREF specifically quantifies the increase in exposure risk due to architectural decisions, we focus on several illustrative architecture variants derived from a video-on-demand (VOD) scenario, chosen for its relevance to real-world system designs, as well as an additional IoT-based example. This enables both a step-by-step demonstration of metric application and a practical reflection of how architecture configurations impact privacy exposure in diverse environments. We keep the same dataset across one case while only changing the data modeling and the architecture configuration. For brevity we only include the resulting values for the assessed metrics. Complete calculations are available at https://ionela-chereja.github.io/PrivacyRiskExpansionFactor/ (accessed on 24 May 2025).

### 4.1. Video-On-Demand (VOD) Ecosystem

This video-on-demand use case presents a simplified and partial view of a video-on-demand system, focusing on defining and including several source systems to illustrate how different architectural choices made throughout the entire data storage ecosystem will impact the calculation of risk exposure metrics. Specific implementation details and project identifiers are intentionally omitted in order to mitigate the potential risk of targeted attacks based on the disclosure of accurate architectural information.

The source systems, collectively referred to as business applications, constitute the core functionality of the data ecosystem. For each subsequent use case, we propose distinct architectural designs aimed at leveraging data from these source systems to generate analytical insights. The proposed architectures vary in the number and complexity of additional layers required to support this objective, while making the same dataset available for analysis. Within the business applications layer, the streaming service constitutes the primary customer-facing system. It maintains a dedicated database to support functionalities such as access provisioning, the presentation of video content and associated metadata, and the recording of user-specific streaming activities. The streaming service interfaces with two distinct back-end systems to enable subscription management: the backend video system and the subscription management system. Data originating from both systems are transmitted to the streaming service and persist independently within their respective storage layers.

### 4.2. VOD—Multi-Layered Ecosystem Architecture Design

**Figure 1:**
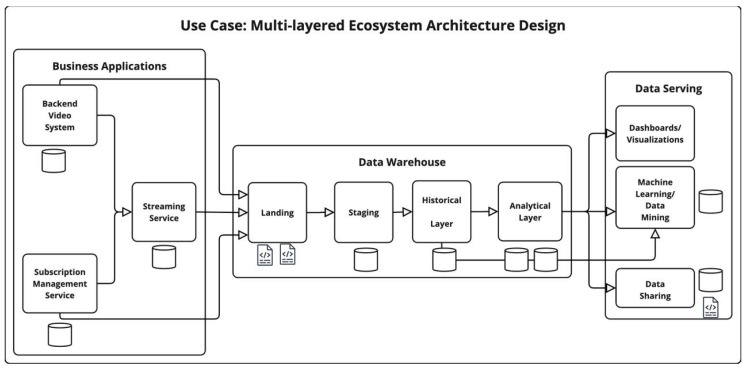
Overview of the Multi-layered Ecosystem (MLE) Architecture Design.

Three primary components are depicted in Figure 1 to illustrate the flow of data between multiple applications and systems. Data originating from each business application is transmitted to the data warehouse layer, which is organized into four sequential steps following a multi-layered enterprise (MLE) data warehouse architecture [65]. Initially, each system independently sends data to the landing storage component, where it is stored in raw files with a semi-structured format. Subsequently, the data undergoes flattening and normalization into a structured format and is loaded into the staging database. To preserve historical records, a further copy of the data is maintained in a dedicated historical layer. Finally, within the data warehouse, data is modeled, aggregated, and centralized into a unified view in the analytical layer. During this final stage, procedures such as encryption and masking are applied to further obfuscate sensitive information.

The final component, the data serving layer, encompasses three distinct processes, all of which can operate in parallel. For the dashboards/visualizations processes, it is assumed that systems directly consume data from the analytical layer; consequently, no additional storage layer is considered for this process. Internal transient storage mechanisms, such as in-memory storage and temporary caching, are excluded from the scope of this work. The remaining two serving processes, machine learning/data mining and data sharing, may each incorporate independent storage layers, either for persisting models used in machine learning/data mining or for maintaining exported data copies in semi-structured files or structured table formats within the data sharing component.

#### 4.2.1. MLE Ecosystem Vulnerability Score

For each of the storage layers described in Figure 1 the vulnerability score is calculated along with the resulting ecosystem vulnerability score. The resulting values are shown, excluding detailed columnar information for brevity, in Table 1.

#### 4.2.2. MLE Privacy Risk Expansion Factor (PREF)

For this use case, we assume the main operations of the business are conducted within the European Union with infrastructure and services designed to comply with regional data governance requirements.

The business applications are hosted on the public cloud, with two primary copies maintained in the main region (West Europe) and an additional replica for disaster recovery purposes [14]. The streaming service disaster recovery replica is located in the United States, while the other two business applications also keep their disaster recovery replicas in a data center in the EU. This creates a difference in the evaluation of the geopolitical location risk (G) metric (see Appendix B).

Each business application is configured with daily backup operations, with the resulting backups hosted in the same geolocation (G = 1.0) and an automated retention policy set to 90 days (R = 0.0). The backend video system and the subscription management system utilize relational database systems, incorporating embedded logging mechanisms that cycle logs, where data marked for deletion remains accessible until it is physically overwritten, resulting in a value of 1.0 for the log accessibility risk metric (L) for all database copies. The streaming service employs a NoSQL database system characterized by high log exposure, which also results in L = 1.0. Across all system components, role-based access control (RBAC) with automated policy enforcement mechanisms is uniformly implemented to ensure secure access management, resulting in a value of 1.0 for the access control scope (A) for the database copies. The backups, however, are stored in a location with shared access, creating a resulting value of A = 1.5 for all business applications.

For all business applications, the main and secondary replicas cannot be removed completely, resulting in a value of D = 1.0. For the streaming service database, the disaster recovery replica hosted outside the EU could be erased; however, the hosting service provides default retention for recoverability, which renders a value of D = 0.5 for this instance. All backup copies could be removed without the possibility to recover the data after removal, resulting in D = 0. Business applications depend on the presence of secondary replicas to enable failovers during updates or unforeseen faults in addition to the disaster recovery replica.

Table 2, Table 3 and Table 4 reflect the values for each of the metrics per copy and layer.

Within the data warehouse and data sharing layers, OLTP storage systems are deployed, each maintaining a single replica dedicated to disaster recovery within the European geolocation; logs for these systems are not accessible and are configured not to retain deleted information after deletion. In addition, the warehouse and sharing databases are configured with automated weekly backups subject to a 30-day retention policy. Data erasure resistance is high across all three business applications, ensuring resilience against data loss, whereas the warehousing and data serving systems demonstrate lower data erasure resistance, given that these systems can be reconstructed if necessary. With the exception of the landing layer, which has been configured for recoverability in case data is removed (as it holds historical raw data from which all the subsequent layers can be reloaded), resulting in D = 0.5 for its secondary copy, and the historical layer, which is configured to maintain a secondary copy that cannot be completely removed due to business constraints (generating D = 1.0), the rest of the warehouse secondary or backup copies result in D = 0.0.

Both the landing and the sharing storage layers result in R = 0.5 for the backup copy, as the retention policies are manually, not automatically, enforced. Details and resulting PREF scores for each of the warehouse and sharing systems are available in Table 5, Table 6, Table 7, Table 8 and Table 9.

An overview of the resulting calculations evaluating PREF are shown in Table 10.

#### 4.2.3. MLE Privacy Exposure Index (PEI)

The overview of the resulting privacy exposure index (PEI) for all layers, including the ecosystem PEI, is available in Table 11.

While the business application layers (streaming service, subscription management service, and backend streaming hosting service) have relatively modest VS scores, they exhibit the highest PREF values. This reflects the operational need for multiple persistent, often deletion-resistant, copies to ensure system availability, failover support, and responsiveness. In contrast, the data warehouse layers, such as the staging, historical, and analytical layers, demonstrate lower PREF scores, as they are less critical to real-time operations, and some can be reconstructed from upstream data, reducing the need for long-term retention.

Importantly, the landing layer stands out with the highest PEI, despite having a lower PREF score than the business applications. This is due to its very high VS, driven by the aggregation of raw and unfiltered data from all source systems. The landing layer thus represents the most exposed component in the architecture, not because of excessive replication, but because of the nature and sensitivity of the data it holds at an early stage. This underscores how architectural context, not just data duplication, plays a pivotal role in privacy exposure and highlights the need for early-stage mitigation strategies.

### 4.3. VOD—Privacy-Aware Ecosystem Architecture Design

**Figure 2:**
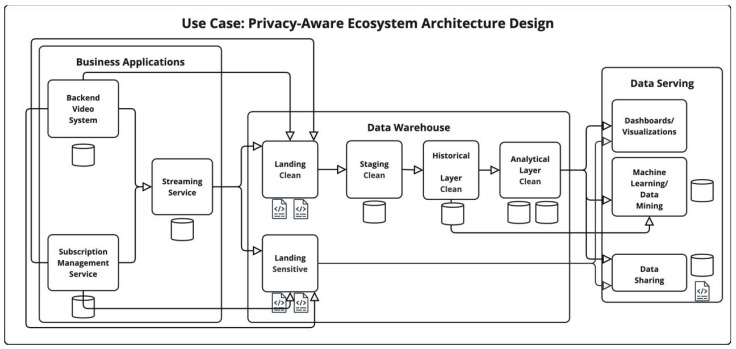
Overview of the Privacy-Aware Ecosystem (PAE) Architecture Design.

In the privacy-aware ecosystem architecture design (PAE), a dedicated warehousing layer is integrated to support analytical and operational needs while ensuring stringent data protection measures. In the initial stage, the traditional landing layer is divided into two distinct storage spaces: a clean landing layer and a sensitive landing layer. The sensitive landing layer stores the complete raw datasets, including sensitive information, whereas the clean landing layer retains only the non-sensitive portions of the incoming data. This architectural separation facilitates a privacy-aware approach by ensuring that all subsequent transformations, processing, and storage operations within the warehousing layer are based exclusively on non-sensitive data. Sensitive data is accessed directly from the sensitive landing layer only when absolutely necessary, thus minimizing the exposure and duplication of sensitive information across other system components. Serving layers consume non-sensitive data by default, with direct interaction with sensitive data being tightly controlled and limited. Although this design introduces additional storage complexity, it enhances data privacy and results in varying levels of data vulnerability depending on the sensitivity and accessibility of each storage space. An overview of the architectural design and data flow is available in Figure 2.

#### 4.3.1. PAE Ecosystem Vulnerability Score

The resulting vulnerability scores for each layer in the privacy-aware architecture are available in Table 12.

#### 4.3.2. PAE Privacy Risk Expansion Factor (PREF)

The resulting PREF for the design of privacy-aware ecosystem (PAE) is shown in Table 13.

#### 4.3.3. PAE Privacy Exposure Index (PEI)

The privacy exposure index (PEI) calculations are shown in Table 14.

### 4.4. VOD—HTAP Ecosystem Architecture Design

In the proposed hybrid transactional/analytical processing (HTAP) architecture, NewSQL storage systems are utilized for all business application systems to enable seamless support for both transactional and analytical workloads within a single database layer. This eliminates the need for additional warehousing storage layers that are traditionally required to provide analytics capabilities for data serving processes [66,67,68]. While this architecture is intended to provide the ability to conduct analysis in real-time, it also lacks historical data.

**Figure 3:**
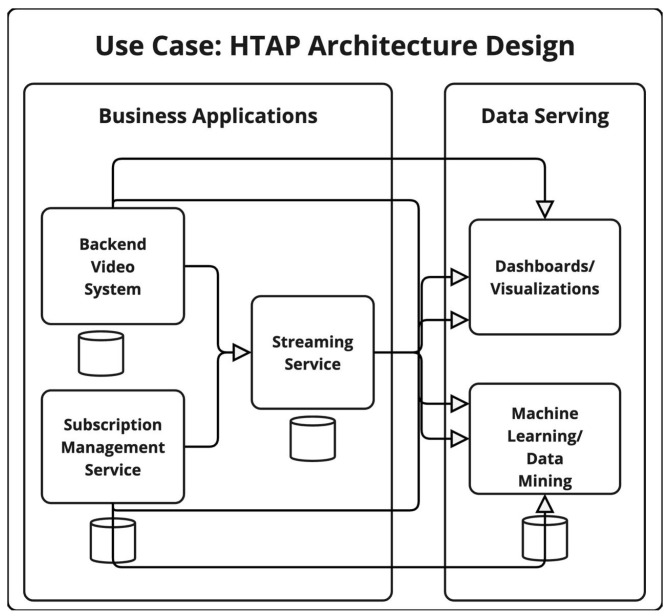
Overview of the HTAP Ecosystem Architecture Design.

The architectural setup follows a configuration similar to the previously introduced designs, maintaining consistent parameters regarding the number of replicas, geographical deployment and hosting [69], backup strategies, access control mechanisms, logging configurations, and retention policies. In alignment with the methodological boundaries established for this study, within which metrics are not evaluated for nonpermanent storage layers, no metric evaluations are conducted for the data serving components in this architecture. An overview of the architecture is shown in Figure 3.

#### 4.4.1. HTAP Ecosystem Vulnerability Score

The calculations for the vulnerability scores of the proposed architecture are available in Table 15.

#### 4.4.2. HTAP Privacy Risk Expansion Factor (PREF)

The resulting PREP for the design of an HTAP ecosystem is shown in Table 16.

#### 4.4.3. HTAP Privacy Exposure Index (PEI)

The privacy exposure index (PEI) calculations are shown in Table 17.

### 4.5. VOD Use Case Evaluation and Discussion

**Figure 4:**
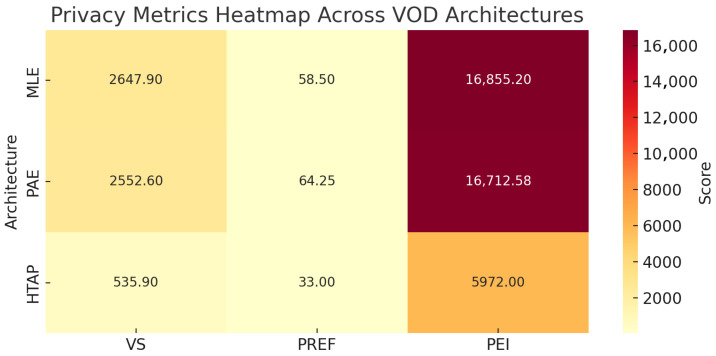
Overview of evaluated metrics for the proposed VOD architectures.

The results of the VOD use case (Figure 4) highlight how architectural choices shape privacy outcomes through measurable impacts on the privacy exposure index (PEI), privacy risk exposure factor (PREF), and vulnerability score (VS). As shown in Table 18, these metrics, while not absolute indicators, offer practical insight into how data modeling and system design affect exposure and privacy vulnerability. MLE exhibits the highest values across all three metrics, reflecting the cost of high-duplication and high-availability configurations. HTAP, with minimal redundancy and centralized processing, shows significantly lower exposure indicators. PAE offers a middle ground, balancing separation and sensitivity management. Collectively, these metrics can serve as analytical and design instruments for assessing how modifications to architectural configurations or dataset characteristics influence data exposure and vulnerability risk, thereby supporting informed decision-making in privacy-oriented system design.

### 4.6. IoT Room Cooling Ecosystem

This IoT room cooling use case presents a set of sensor data commonly found in smart environmental cooling systems. The columns used in this particular use case are the controller, sensor, and room IDs, along with the temporal data and sensor values. Sensor data is transmitted to cloud storage to be persisted and analyzed.

### 4.7. IoT—Multi-Layered Environmental Sensor Ecosystem Architecture Design

**Figure 5:**
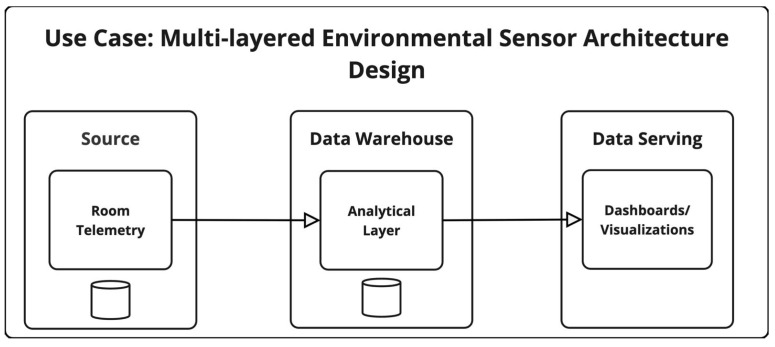
Overview of Multi-Layered Environmental Sensor (MLES) Ecosystem Architecture Design.

The architecture of this IoT environmental sensor system involves multiple stages of data handling that influence its privacy exposure. Sensor data, including identifiers, environmental readings, and occupancy metrics, are first collected locally and transmitted to a centralized time series database in the cloud for real-time storage. From there, the data is duplicated into a separate data analysis database where it is aggregated and processed for insights on smart cooling and room usage. The source database for the room telemetry is configured to have two replicas and one daily backup with automated retention, whereas the analytical layer is configured to have three replicas (this includes a disaster recovery replica) and a daily backup with strict enforcement of retention. A visual overview of the MLES architecture is available in Figure 5.

#### 4.7.1. MLES Ecosystem Vulnerability Score

The resulting vulnerability score for the persistent data layer in the multi-layered environmental sensor architecture is available in Table 19.

#### 4.7.2. MLES Privacy Risk Expansion Factor (PREF)

The resulting PREP for the proposed design of the multi-layered environmental sensor ecosystem (MLES) is shown in Table 20.

#### 4.7.3. MLES Privacy Exposure Index (PEI)

The privacy exposure index (PEI) calculations are shown in Table 21.

### 4.8. IoT—HTAP Environmental Sensor Ecosystem Architecture Design

**Figure 6:**
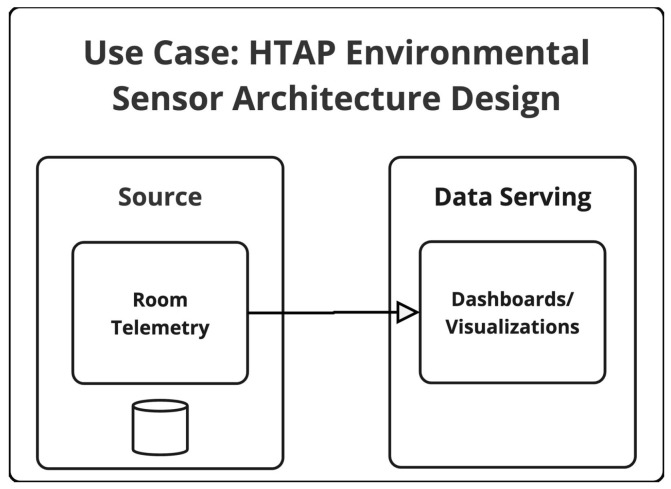
Overview of HTAP Environmental Sensor (HTAPS) Ecosystem Architecture Design.

The proposed architecture of this IoT environmental sensor system involves only one stage of data handling. Data is transmitted to a centralized cloud-based HTAP (hybrid transactional/analytical processing) storage system. This HTAP database supports both high-throughput ingestion of real-time sensor streams and low-latency analytical queries, enabling direct access for monitoring dashboards and analytical applications without the need for duplicating or moving data into a separate analytical layer. By consolidating transactional and analytical processing within the same infrastructure, the architecture minimizes data replication, reduces architectural exposure points, and supports efficient privacy risk management. An overview of the proposed HTAP architecture is available in Figure 6.

#### 4.8.1. HTAPS Ecosystem Vulnerability Score

The resulting vulnerability score for the persistent data layer in the environmental sensor architecture is available in Table 22.

#### 4.8.2. HTAPS Privacy Risk Expansion Factor (PREF)

The resulting PREP for the proposed design of the HTAP architecture is shown in Table 23.

#### 4.8.3. HTAPS Privacy Exposure Index (PEI)

The privacy exposure index (PEI) calculations are shown in Table 24.

### 4.9. IoT Use Case Evaluation and Discussion

**Figure 7:**
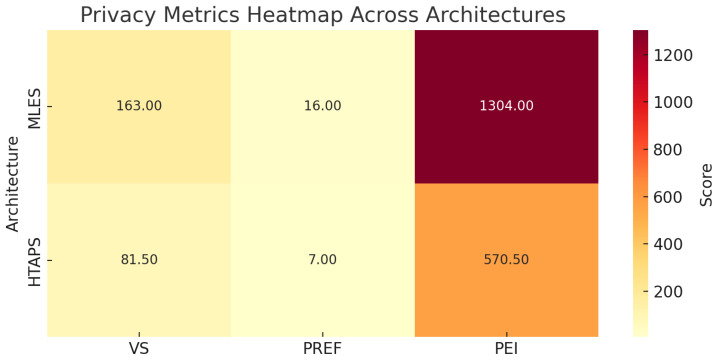
Overview of evaluated metrics for the proposed IoT architectures.

The analysis in Table 25 shows how PREF and PEI offer practical metrics for informing architectural decisions. While not absolute indicators, they help quantify privacy and exposure risks. As shown in Figure 7, MLES scores higher due to data replication and backups, reflecting greater exposure despite benefits like high availability and disaster recovery. HTAP, with lower PREF and PEI, reduces risk through minimal data movement, though it may sacrifice analytical flexibility and performance. These metrics support more informed trade-offs between resilience, efficiency, and privacy in architectural design.

## 5. Results

The computational analysis across the use case scenarios shows that even when data types are uniform within an architectural layer, exposure risk varies significantly based on architectural design. In classic warehousing setups, where data is repeatedly copied across multiple layers, the vulnerability score (VS) rises due to increased exposure points. While VS highlights where sensitive data resides, the privacy risk expansion factor (PREF) adds context by capturing how architectural configurations such as duplication, persistence, and geolocation amplify exposure. Combined, they form the privacy exposure index (PEI), offering a quantifiable, though not absolute, measure of relative exposure risk.

The comparative analysis highlights how PREF and PEI provide actionable insights into the privacy trade-offs of architectural choices. For instance, privacy-aware designs reduce overall exposure by limiting the propagation of sensitive data, reflected in a lower PEI. However, their layered structure sustains a moderate PREF, indicating residual architectural complexity. Conversely, the two proposed HTAP configurations achieve the lowest PREF and PEI values by minimizing data replication and movement, demonstrating an efficient, low-exposure approach. However, while these architectures can be highly effective in reducing exposure and data duplication, they may be less suitable for systems requiring extensive historical data retention or those with intensive analytical workloads, where HTAP’s unified processing model could introduce performance constraints.

While these metrics do not measure exposure in absolute terms, they enable early-stage, data-agnostic evaluations of design alternatives. As illustrated in Table 18 and Table 25, PREF and PEI help quantify relative risk expansion and data exposure and guide the development of privacy-conscious architectures.

## 6. Discussion

The results presented in this work demonstrate the potential of the proposed methodology to quantitatively assess exposure risks across varying data architectures and types through metrics such as the privacy risk expansion factor (PREF), the vulnerability score (VS), and the privacy exposure index (PEI). These metrics offer a structured and objective foundation to evaluate how architectural and data-handling choices affect the exposure and propagation of sensitive information across systems.

However, the framework also has several known limitations. First, it operates under the assumption of static inputs—governance, access, and replication parameters are treated as fixed, while in practice these attributes evolve over time. Without dynamic and continuous recalibration, this can lead to inaccuracies in the evaluated risk profile. Additionally, the current metric ranges and weightings are heuristic, lacking empirical calibration against historical incident data or observed breach patterns.

Future work could address these challenges through multiple avenues. Incorporating time-aware or dynamic variants of PREF and PEI could enable the framework to reflect evolving system topologies and retention behaviors. Integration with automated tools for architecture analysis and data discovery would support continuous risk assessment and reduce the manual burden of computation. Further, future work could focus on empirically calibrating these values through real-world breach datasets, expert risk assessment surveys, and controlled audit-based validations. As privacy threats grow in complexity, expanding the framework to include inter-copy correlation modeling and attacker profiling could be a good avenue for capturing the full scope of system vulnerability.

Furthermore, future research could address the challenges that arise from dynamic and distributed data ecosystems, where components, data flows, and exposures constantly evolve. Developing comprehensive dashboards and visualization frameworks to interpret real-time metrics would also be instrumental in translating technical assessments into actionable insights.

## 7. Conclusions

This work introduces two new privacy assessment metrics, the privacy risk expansion factor (PREF) and the privacy exposure index (PEI), which build on and complement existing approaches to data vulnerability, particularly the previously established vulnerability score (VS) for data content. Together, these metrics provide a more comprehensive framework for evaluating privacy risks in data architecture designs. PREF captures the impact of architectural complexity on the spread and exposure of data, while PEI integrates content vulnerability with architectural exposure into a single, interpretable index. By combining these aspects, the methodology supports a more systematic identification of privacy risks across different layers and stages of data processing.

The contribution of this work lies in formalizing the interplay between data contents and architectural configurations that further expose the data contents, offering objective metrics that can guide the design and evaluation of privacy-aware systems from an early stage. The proposed approach facilitates comparative analysis between architectures, highlighting how different design choices affect privacy exposure. Moreover, it lays the groundwork for future research into dynamic, real-time privacy risk monitoring and AI-driven privacy assessment tools. By extending static assessments with adaptable metrics, this framework supports the development of more resilient and privacy-preserving data ecosystems.

## Figures and Tables

**Table 1:** Vulnerability score overview for the multi-layered ecosystem architecture.

Data Layer	Vulnerability Score
Streaming Service	212.4
Subscription Management Service	164.0
Backend Streaming Hosting Service	159.5
Landing	559.7
Staging	535.9
Historical	524.7
Analytical	285.2
Sharing	206.5
Ecosystem	2647.9

**Table 2:** Streaming service privacy risk expansion factor.

Copy Type	G	A	R	D	L	PREF
Main Copy	1.00	1.00	0.00	1.00	1.00	3.00
Secondary Copy	1.00	1.00	0.00	1.00	1.00	3.00
Disaster Recovery Copy	2.00	1.00	0.00	0.50	1.00	5.00
Daily Backup	1.00	1.50	0.00	0.00	0.00	1.50
Total PREF						12.50

**Table 3:** Subscription management service privacy risk expansion factor.

Copy Type	G	A	R	D	L	PREF
Main Copy	1.00	1.00	0.00	1.00	1.00	3.00
Secondary Copy	1.00	1.00	0.00	1.00	1.00	3.00
Disaster Recovery Copy	1.00	1.00	0.00	1.00	1.00	3.00
Daily Backup	1.00	1.50	0.00	0.00	0.00	1.50
Total PREF						10.50

**Table 4:** Backend streaming hosting service privacy risk expansion factor.

Copy Type	G	A	R	D	L	PREF
Main Copy	1.00	1.00	0.00	1.00	1.00	3.00
Secondary Copy	1.00	1.00	0.00	1.00	1.00	3.00
Disaster Recovery Copy	1.00	1.00	0.00	0.50	1.00	2.50
Daily Backup	1.00	1.50	0.00	0.00	0.00	1.50
Total PREF						10.00

**Table 5:** Landing privacy risk expansion factor.

Copy Type	G	A	R	D	L	PREF
Main Copy	1.00	1.00	0.00	1.00	0.00	2.00
Secondary Copy	1.00	1.00	0.00	0.50	0.00	1.50
Weekly Backup	1.00	1.50	0.50	0.00	0.00	2.25
Total PREF						5.75

**Table 6:** Staging privacy risk expansion factor.

Copy Type	G	A	R	D	L	PREF
Main Copy	1.00	1.00	0.00	1.00	0.00	2.00
Secondary Copy	1.00	1.00	0.00	0.00	0.00	1.00
Weekly Backup	1.00	1.50	0.00	0.00	0.00	1.50
Total PREF						4.50

**Table 7:** Historical privacy risk expansion factor.

Copy Type	G	A	R	D	L	PREF
Main Copy	1.00	1.00	0.00	1.00	0.00	2.00
Secondary Copy	1.00	1.00	0.00	1.00	0.00	2.00
Weekly Backup	1.00	1.50	0.00	0.00	0.00	1.50
Total PREF						5.50

**Table 8:** Analytical privacy risk expansion factor.

Copy Type	G	A	R	D	L	PREF
Main Copy	1.00	1.00	0.00	1.00	0.00	2.00
Secondary Copy	1.00	1.00	0.00	0.00	0.00	1.00
Weekly Backup	1.00	1.50	0.00	0.00	0.00	1.50
Total PREF						4.50

**Table 9:** Sharing privacy risk expansion factor.

Copy Type	G	A	R	D	L	PREF
Main Copy	1.00	1.00	0.00	1.00	0.00	2.00
Secondary Copy	1.00	1.00	0.00	0.00	0.00	1.00
Manual Backup	1.00	1.50	0.50	0.00	0.00	2.25
Total PREF						5.25

**Table 10:** PREF score overview for the multi-layered ecosystem architecture.

Data Layer	PREF Score
Streaming Service	12.5
Subscription Management Service	10.5
Backend Streaming Hosting Service	10.0
Landing	5.75
Staging	4.5
Historical	5.5
Analytical	4.5
Sharing	5.25
Ecosystem	58.5

**Table 11:** PEI score overview for the multi-layered ecosystem architecture.

Data Layer	VS	PREF	PEI
Streaming Service	212.4	12.5	2655.0
Subscription Management Service	164.0	10.5	1722.0
Backend Streaming Hosting Service	159.5	10.0	1595.0
Landing	559.7	5.75	3218.275
Staging	535.9	4.5	2411.55
Historical	524.7	5.5	2885.85
Analytical	285.2	4.5	1283.4
Sharing	206.5	5.25	1084.125
Ecosystem	2647.9	58.5	16,855.2

**Table 12:** Vulnerability score overview for the Privacy-Aware Ecosystem Architecture.

Data Layer	Vulnerability Score
Streaming Service	212.4
Subscription Management Service	164.0
Backend Streaming Hosting Service	159.5
Landing Sensitive	559.7
Landing Clean	368.5
Staging Clean	350.3
Historical Clean	350.3
Analytical Clean	181.4
Sharing	206.5
Ecosystem	2552.6

**Table 13:** PREF score overview for the Privacy-Aware Ecosystem Architecture.

Data Layer	PREF
Streaming Service	12.5
Subscription Management Service	10.5
Backend Streaming Hosting Service	10.0
Landing Sensitive	5.75
Landing Clean	5.75
Staging Clean	4.5
Historical Clean	5.5
Analytical Clean	4.5
Sharing	5.25
Ecosystem	64.25

**Table 14:** Privacy exposure index (PEI) overview for the Privacy-Aware Ecosystem Architecture.

Data Layer	VS	PREF	PEI
Streaming Service	212.4	12.5	2655.0
Subscription Management Service	164.0	10.5	1722.0
Backend Streaming Hosting Service	159.5	10.0	1595.0
Landing Sensitive	559.7	5.75	3218.275
Landing Clean	368.5	5.75	2118.875
Staging Clean	350.3	4.5	1576.35
Historical Clean	350.3	5.5	1926.65
Analytical Clean	181.4	4.5	816.3
Sharing	206.5	5.25	1084.125
Ecosystem	2552.6	64.25	16,712.575

**Table 15:** Vulnerability score overview for the HTAP architecture.

Data Layer	Vulnerability Score
Streaming Service	212.4
Subscription Management Service	164.0
Backend Streaming Hosting Service	159.5
Ecosystem	535.9

**Table 16:** PREF score overview for the HTAP architecture.

Data Layer	PREF
Streaming Service	12.5
Subscription Management Service	10.5
Backend Streaming Hosting Service	10.0
Ecosystem	33.0

**Table 17:** Privacy exposure index (PEI) overview for the HTAP architecture.

Data Layer	VS	PREF	PEI
Streaming Service	212.4	12.5	2655.0
Subscription Management Service	164.0	10.5	1722.0
Backend Streaming Hosting Service	159.5	10.0	1595.0
Ecosystem	535.9	33.0	5972.0

**Table 18:** Privacy risk trade-off matrix across architectural designs.

Architecture	Design Trade-Offs	Privacy Risk Summary
MLE	High duplication, persistent storage across layers	High PREF and highest PEI, especially in the landing and historical layers
PAE	Layer separation, minimized duplication, clean/sensitive data split	Highest PREF, lower PEI, improved downstream privacy posture
HTAP	Minimal redundancy, centralized processing, real-time access	Lowest PREF, lowest PEI centralized in single processing tier

**Table 19:** Vulnerability score overview for the Multi-Layered Environmental Sensor Architecture.

Data Layer	Vulnerability Score
Room Telemetry	81.5
Analytical Layer	81.5
Ecosystem	163.0

**Table 20:** PREF score overview for the Multi-Layered Environmental Sensor Architecture.

Data Layer	PREF
Room Telemetry	7.0
Analytical Layer	9.0
Ecosystem	16.0

**Table 21:** Privacy exposure index (PEI) overview for the Multi-Layered Environmental Sensor Architecture.

Data Layer	VS	PREF	PEI
Room Telemetry	81.5	7.0	570.5
Analytical Layer	81.5	9.0	733.5
Ecosystem	163	16.0	1304.0

**Table 22:** Vulnerability score overview for the HTAP Environmental Sensor Architecture.

Data Layer	Vulnerability Score
Room Telemetry	81.5
Ecosystem	81.5

**Table 23:** PREF score overview for the HTAP Environmental Sensor Architecture.

Data Layer	PREF
Room Telemetry	7.0
Ecosystem	7.0

**Table 24:** Privacy exposure index (PEI) overview for the HTAP Environmental Sensor Architecture.

Data Layer	VS	PREF	PEI
Room Telemetry	81.5	7.0	570.5
Ecosystem	81.5	7.0	570.5

**Table 25:** Privacy risk trade-off matrix across iot architectural designs.

Architecture	Design Trade-Offs	Privacy Risk Summary
MLES	Multi-stage data handling, separate analytical layer, multiple replicas and backups	Increased exposure from duplication and retention layers; higher PREF and PEI due to redundancy across tiers
HTAP	Single-stage HTAP system, unified transactional and analytical processing	Minimal replication and movement; lower PREF and PEI; reduced vulnerability surface

## Data Availability

The raw data supporting the conclusions of this article will be made available by the authors upon request.

## Abbreviations

The following abbreviations are used in this manuscript:

AI	Artificial intelligence
CCPA	California Consumer Privacy Act
CVSS	Common vulnerability scoring system
ENCD	Encoded data metric
ENCR	Encrypted data metric
EXPL	Exploitability data metric
GDPR	General Data Protection Regulation
HPI	Personal information data metric
HSI	Sensitive/secret information data metric
HNS	Nested sensitive information data metric
HUS	Unstructured sensitive/secret information data metric
HTAP	Hybrid transactional/analytical processing
IoT	Internet of Things
LLM	Large language model
MASK	Masked data metric
PbD	Privacy by design
PEI	Privacy exposure index
PET	Privacy-enhancing technologies
PII	Personal identifiable information
PREF	Privacy risk expansion factor
SMPC	Secure multi-party computation
TEE	Trusted execution environment
VS	Vulnerability score

## Appendix A. Access Control Scope Score

The access control scoring metric (*A*) provides a structured approach to evaluating the strength of access control mechanisms within a system. Lower values indicate stronger, more granular and auditable enforcement, while higher values reflect weaker or absent controls. While the Table A1 offers representative categories, assessors may assign intermediate scores to capture edge cases or hybrid implementations, provided they stay within the defined bounds. This flexibility supports more precise evaluation across diverse architectural contexts.

**Table A1:** *A* Access control scope score.

Value	Access Control Level	Examples
1.0	Strong Access Control	Role-based access control (RBAC) with automated enforcement, attribute-based access control (ABAC), database-level row/column restrictions, just-in-time (JIT) access with expiry and audit trails
1.5	Moderate or Weak Access Control	Single access role for multiple users, stale or unrevoked access rights, lack of context-aware or fine-grained policies
2.0	Minimal or No Access Control	Shared or hardcoded credentials, no access logging or monitoring, anonymous or unrestricted access

## Appendix B. Geopolitical Location Risk Score

The geopolitical location risk score (*G*) offers a structured lens for assessing the privacy and legal exposure associated with where data is physically and jurisdictionally hosted. Lower values represent low-risk environments, typically jurisdictions with strong privacy laws and regulatory alignment while higher values denote settings with weak protections or legal conflicts. While the categories in Table A2 provide clear guidance, assessors may apply intermediate scores to account for hybrid or evolving legal contexts that are relevant to the internal policies and jurisdictional considerations.

**Table A2:** *G* Geopolitical location risk score.

Value	Geopolitical Risk Level	Example
1.0	Low Risk	Data hosted in jurisdictions with strong privacy robust enforcement, and strong alignment with the data owner’s legal and operational base. (on premises, private, or public cloud: legally under full control of the data owner’s organization)
2.0	High Risk	Data located in or accessible from jurisdictions with extraterritorial surveillance laws, weak privacy enforcement, or conflicting legal systems. Providers in non-adequate countries without contractual/legal protections.

## Appendix C. Retention and Life Cycle Policy Compliance Score

Table A3 lists possible values for retention and life cycle policy compliance (*R*). *R* represents the extent to which the data copy adheres to defined data retention and deletion policies. Even if data is erasable, retention policies dictate how long the copy persists. If there is no automated expiration, the copy might live indefinitely. Higher values indicate weaker or absent policy enforcement, increasing the risk of prolonged or unnecessary data storage and exposure. The value 0 can be used for cases where automatic retention policies are irrelevant, such as for primary copies stored in operational data systems, under the ‘Not Applicable’ category.

**Table A3:** *R* Retention and life cycle policy compliance score.

Value	Policy Enforcement Level	Example
0.0	Strictly enforced or Not Applicable	Automated deletion based on policy; retention schedules enforced by system; temporary or read-only data copies not subject to policy due to non-persistence; Not Applicable
0.5	Partially enforced policy	Retention policy defined but dependent on manual enforcement; risk of human error or delays in deletion
1.0	Unclear or missing policy	No defined retention schedule; data retained indefinitely or deleted without auditability

## Appendix D. Data Erasure Resistance Score

Table A4 lists possible values for data erasure resistance (*D*). *D* represents how easily an entire copy of the data is securely and verifiably erased. This takes into account whether a database node, backup, or distributed replica can be completely wiped or destroyed. Measurement at the copy level captures both physical and logical persistence, which is especially relevant in the context of incident response and regulatory compliance.

**Table A4:** *D* Data erasure resistance score.

Value	Erasure Resistance Level	Example
0.0	Low erasure resistance	Data copies can be fully deleted; no residual data remains; complete erasure confirmed
0.5	Moderate erasure resistance	Erasure possible but incomplete; copy may be recoverable through shadow copies or incomplete deletion of replica
1.0	High erasure resistance	Copies are immutable or persistent by design; includes backups, unmodifiable snapshots, or retention-locked archives

## Appendix E. Log Accessibility Risk Score

Log accessibility risk (*L*) assesses the extent to which system logs might expose sensitive data or internal operations. Lower scores indicate well-protected logging practices, while higher scores reflect risky configurations like unencrypted logs, overexposure, or excessive retention. Although Table A5 is not exhaustive, it offers a structured basis for evaluating log-related risks. Assessors may assign intermediate values to capture detailed scenarios or edge cases more accurately.

**Table A5:** *L* Log accessibility risk score.

Value	Risk Category	Example
0.0	Low Risk	Logs encrypted at rest and in transit, access-controlled platforms, log redaction of sensitive data, short retention with auto-deletion
0.5	Medium Risk	Partial encryption (e.g., only at rest), basic access control with limited auditing, inconsistent redaction, moderate retention periods, occasional inclusion of metadata like IPs or user IDs
1.0	High Risk	Logs in plain text, broad or unmonitored access, over-retention or missing expiration policy, debug/trace logs exposing internals, soft deletion allowing sensitive data recovery

## Appendix F. Encryption Algorithms Score

Table A6 lists example values for commonly used data encryption algorithms. While not exhaustive, it serves as a quick reference rather than a comprehensive evaluation of all existing methods. Stronger, more secure algorithms are assigned values closer to 1.0 (top of the scale), whereas weaker or easily compromised methods trend toward 2.0, with “No Encryption” representing the upper bound of risk.

**Table A6:** Encryption algorithm score.

Value	Algorithm
1.00	AES
1.10	ECC
1.20	3DES
1.30	
1.40	
1.50	RSA 1024
1.60	
1.70	
1.80	SHA-1
1.90	MD5
2.00	No Encryption

## Appendix G. Exploitability Score

Exploitability (Table A7) measures the potential for a malicious actor to misuse the data for illicit purposes. The assigned values are derived through relative scaling, comparing each data element with the most- and least-critical in terms of sensitivity and misuse potential.

**Table A7:** Data exploitability score.

Value	Contents	Examples
1.00	Data with no direct or indirect link to specific persons, groups of persons, families, or neighborhoods	Environmental data (temperature)
1.10		
1.20	Data with a weak potential link to persons	Land GPS coordinates that might be private property
1.30		
1.40	Data with a strong potential link to persons	Anonymous information about houses that might be used to locate them (price or neighborhood)
1.50	Non-exploitable personal information (that might still have potential economic value), i.e., anonymized medical data	Height, hair color, eye color, blood test results, or preferences (food, beverages, or deserts)
1.60		
1.70	Anonymized medical data with demographic/personal information	Cancer screening results or AIDS patient data
1.80	Low-exploitable personal information (that is easy to change or cannot be used to locate the person in real life)	Email (but no password!) or mobile number
1.90	Non-anonymized medical data	
2.00	Highly-exploitable and secret personal information, identity information, or national security-related information	Complete name, social security number, home address, home phone number, home building properties (e.g., intercom code), intimate personal photos, personal messages, sexual preferences, passwords, religious views, or political affiliation
